# Clinical effect of Danshen decoction in patients with heart failure: A systematic review and meta-analysis of randomized controlled trials

**DOI:** 10.1371/journal.pone.0284877

**Published:** 2023-05-05

**Authors:** Ziyi Li, Mengnan Liu, Mingtai Chen, Gang Luo, Jiao Wu, Maryam Mazhar, Fang Yang, Yu Zheng, Hao Wu, Qibiao Wu, Sijin Yang

**Affiliations:** 1 School of Clinical Medicine, Southwest Medical University, Luzhou, P.R. China; 2 National Traditional Chinese Medicine Clinical Research Base and Department of Cardiovascular Medicine, The Affiliated Traditional Chinese Medicine Hospital of Southwest Medical University, Luzhou, P.R. China; 3 Faculty of Chinese Medicine and State Key Laboratory of Quality Research in Chinese Medicine, Macau University of Science and Technology, Macau, P.R. China; 4 Department of Cardiovascular Disease, Shenzhen Traditional Chinese Medicine Hospital, Shenzhen, PR China; 5 Chengdu University of Traditional Chinese Medicine, Chengdu, P.R. China; University of Florida, UNITED STATES

## Abstract

**Background:**

The incidence of heart failure (HF) is increasing year by year, posing a great threat to human health. Although pharmacotherapy has been able to significantly prolong patient survival, pharmacotherapy for HF still has limitations due to its complex pathogenesis and considerable individual variability, there is a great need to explore complementary and alternative therapies to slow down the progression of HF. Danshen decoction is used to treat several cardiovascular diseases including HF, but the efficacy of stabilization is uncertain. This meta-analysis evaluated the clinical efficacy of Danshen Decoction for the treatment of HF.

**Methods:**

The registration number assigned to this meta-analysis on the PROSPERO platform is CRD42022351918. Four databases were searched, and randomized controlled trials (RCTs) of Danshen decoction combined with conventional treatment (CT) of HF were screened, CT included medical treatments other than Danshen Decoction to which the patient was treated (including but not limited to angiotensin converting enzyme inhibitor, angiotensin receptor blocker, angiotensin receptor neprilysin inhibitors, β-blockers, diuretics, mineralcorticoid recept antagonist etc.). The clinical efficacy rate (CER), left ventricular ejection fraction (LVEF), left ventricular end-diastolic dimension (LVEDD), left ventricular end-systolic diameter (LVESD), brain natriuretic peptide (BNP), N-terminal pro-B type natriuretic peptide (NT-proBNP) and hypersensitive C-reactive protein (hs-CRP) were included as outcome indicators. The GRADE grading scale was used to grade the above indicators. The Cochrane risk-of-bias tool and the Jadad quality scale were used to assess the methodological quality of RCTs. Finally, RevMan V.4.5 software was used for data synthesis, 95% confidence intervals (CI) for dichotomous data, risk ratios (RR), and mean differences (MD) for continuous variables were calculated, Chi-square and *I*^2^ were used for heterogeneity assessment.

**Results:**

Nine RCTs with a total of 855 patients were included in this study, and all included RCTs had a low overall quality risk of bias and high quality of reported information. The results of the meta-analysis showed that compared with the use of CT, CER (%) was significantly improved due to Danshen decoction combined with CT (MD = 3.95, 95% CI [2.58, 6.04], *P* < 0.00001), LVEF (%) was significantly improved (MD = 5.46, 95% CI [5.32, 5.60], *P* < 0.00001), LVEDD (mm) was significantly reduced (MD = -5.27, 95% CI [-6.21, -4.32], *P* < 0.00001), LVESD (mm) was significantly reduced (MD = -4.60, 95% CI [-5.87, -3.32], *P* < 0.00001), BNP (pg/mL) was significantly reduced (MD = -88.61, 95% CI [-121.98, -55.24], *P* < 0.00001), NT-proBNP (pg/mL) was significantly decreased (SMD = -3.33, 95% CI [-5.92, -0.73], *P* = 0.01), hs-CRP (mg/L) was significantly decreased (MD = -2.73, 95% CI [-4.11, -1.34], *P* = 0.0001). The quality of the GRADE evidence for all outcomes was moderate to low and no RCTs reported adverse events.

**Conclusion:**

Our research demonstrates that Danshen decoction is an effective and safe treatment option for HF. Nevertheless, considering the limitations of methodological and the quality of RCTs, more rigorous, large-scale, multicenter randomized clinical trials are needed to further evaluate the efficacy and safety of Danshen decoction in the treatment of HF patients.

## Introduction

Heart failure (HF) is considered a new epidemic due to its increasing incidence and long duration [1]. In *Summary of China Cardiovascular Health and Disease Report 2021*, it is reported that the number of cardiovascular patients in China is about 330 million, of which 12.1 million are HF [2, 3], and the incidence rate increases with age [4]. The primary diseases of HF include myocardial infarction, myocarditis, hypertension and various risk factors that increase peripheral circulatory resistance leading to HF [5]. The main treatment methods include drug therapy and surgery. Pharmacotherapy includes angiotensin converting enzyme inhibitor, angiotensin receptor blocker, angiotensin receptor neprilysin inhibitors, β-blockers, diuretics, mineralcorticoid recept antagonist etc. [6], and surgical treatment includes heart transplantation and placement of cardiac assist devices [7, 8]. However, long-term drug treatment may cause side effects such as bradycardia, hypotension, and arrhythmia [9]. Surgical treatment also renders greater safety risks and impose strict restrictions on patients’ conditions [10]. The pathogenesis of HF is quite complex and there is considerable individual heterogeneity of the disease, there is a great need to explore complementary and alternative therapies to slow down the progression of HF [11].

Traditional Chinese medicine (TCM) has a history of more than 2,000 years in the treatment of HF, it was first recorded in the Yellow Emperor’s Canon of Internal Medicine (26 B.C.) [12, 13], and various prescriptions have been widely recognized for their therapeutic effects on HF after long-term clinical practice (Including Zhenwu decoction, Linggui Zhugan decoction, Wuling Powder, and Danshen decoction etc.) [14]. Danshen decoction is composed of *Salvia miltiorrhiza* Bge., *Amomum villosum* Lour, and *Santalum album* L [15, 16], and the details are shown in Table 1. *Shifang Gekuo* (AD 1801) recorded that it was mainly used for treating heartache and epigastric pain. At present, a large number of clinical and experimental studies have shown that Danshen decoction plays an important auxiliary function in the treatment of HF, and its therapeutic effects include improving cardiac function (CF), reducing peripheral vascular resistance, alleviating inflammation, etc. [17]. Until now, most of the published studies have witnessed consistent efficacy of Danshen decoction combined conventional treatment in the treatment of various cardiovascular diseases [18]. However, there is still a lack of evidence-based medical evaluation to prove the safety and efficacy of Danshen decoction in clinical treatment. Therefore, we conducted this meta-analysis to form an opinion on the treatment of HF with Danshen decoction and consider its prospects as an complementary and alternative therapy [19, 20].

**Table 1:** The composition of Danshen decoction and function of each herb.

Chinese name	Pharmaceutical name	Family	Dosage	Function in Chinese medicine
Danshen	*Salvia miltiorrhiza* Bge.	Lamiaceae	96 g	To promotes blood circulation and removes blood stasis, calm the heart and relieves pain, cools blood and eliminates carbuncle.
Tanxiang	*Santalum album* L.	Sandalwood	16 g	Promoting the circulation of *qi*, warming the middle and appetiting and pain.
Sharen	*Amomum villosum* Lour	Ginger	16 g	To dissolving dampness and appetizing, warming the spleen and stopping diarrhea, regulating *qi* and preventing miscarriage.

In this paper, all studies related to Danshen decoction were searched, and inclusion-exclusion criteria was formulated to screen out all the eligible clinical studies. Afterwards, Review Manager 5.4 software was used to conduct a systematic and comprehensive review of the nine randomized controlled trials (RCTs). The clinical efficacy rate (CER) of Danshen decoction combined with conventional treatment (CT) in patients with HF, as well as the improvement of Danshen decoction combined with CT on CF, serological diagnostic indicators such as brain natriuretic peptide (BNP) [21], N-terminal pro-B type natriuretic peptide (NT-proBNP) and hypersensitive C-reactive protein (hs-CRP) in patients with HF were evaluated [22]. This study provides valuable evidence-based support for the clinical application of Danshen decoction in the treatment of cardiovascular diseases [23, 24].

## Methods

This study used the preferred reporting item of the guidelines for systematic reviews and meta-analyses (PRISMA) and all research procedures were completed in accordance with PRISMA requirements [25, 26], and this study has been proofread by PRISMA 2020 Checklist, as shown in S1 Table. The details of our research have been registered in PROSPERO (https://www.crd.york.ac.uk/PROSPERO/) [27] with the registration number: CRD42022351918. The details of all studies refer to our previously published protocol [28].

### Search strategy

Four databases, PubMed (https://pubmed.ncbi.nlm.nih.gov/), CNKI (https://www.cnki.net/), VIP (http://www.cqvip.com/) and Wanfang (https://www.wanfangdata.com.cn/) were used for retrieval, the retrieval time is from the establishment of the database to October 2022. The following keywords were used for individual or joint searches: randomized controlled trial, clinical controlled experiment, clinical observation, Danshen decoction, cardiovascular system, coronary heart disease, heart failure, ischemia-reperfusion injury, angina pectoris, heart valve disease, hypertension, pericardial disease, endocarditis, cardiac arrest, and sudden cardiac death. According to the above search method, RCTs related to the treatment of HF with Danshen decoction were manually screened and included in this study. The search strategy was presented as follows by taking PubMed as an example (S2 Table).

### Type of studies

Inclusion criteria: (a) RCTs were related to Danshen decoction. (b) All included patients had to have one of the diagnoses with HF, Grading of disease should refer to the 2022 AHA/ACC/HFSA guideline for the management of heart failure (defined as B, C, or D stage) [29], or using the New York Heart Association (NYHA) grade to define cardiac function (defined as II, III or IV). (c) CER was defined as the primary outcome of RCTs, and the secondary outcomes were echocardiography and serological indicators. Exclusion criteria: (a) Duplicate articles. (b) Literature, other than English or Chinese language. (c) Grey literature and unpublished literature with unclear experimental data or incorrect results.

### Data extraction

Two investigators (Ziyi Li and Mengnan Liu) independently screened titles and abstracts that might match studies. The full text of possible studies was then searched and summarized according to previously established inclusion-exclusion criteria. Data from clinical studies were then extracted independently by two investigators (Gang Luo and Hao Wu), including the title of each study, name of the first author, type of design, time of publication, sample size, grouping, mean age of patients included, primary diagnosis and diagnostic criteria, intervention measures in experimental group and control group, clinical feature and the result of laboratory detection, the dosage of administration, observation time, adverse drug reactions and reasons for withdrawal. Any discrepancies were discussed with the third investigator (Yu Zheng).

### Methodological quality assessment

According to the Cochrane Collaboration Tool, the methodological quality of each included trial was scored by 2 investigators (Gang Luo and Yu Zheng). It consisted of seven areas: adequate sequence generation, hidden assignments, blinding participants and people, incomplete result data, selective reporting, and other deviations. There were three levels used to assess the quality of the methodology: “low risk of bias” (+), “high risk of bias” (-), and “unclear risk of bias” (?). If necessary, differences were discussed with a third investigator (Hao Wu) to reach a consensus conclusion.

Jadad scale tool was used to check the methodological quality of each included RCTs by two investigators (Gang Luo and Yu Zheng), the following four aspects were included: random sequence generation, randomization concealment, blinding, retreat and withdrawal [30]. Three levels were used to assess the quality of the method: Highly Compliant (2 points), Moderately Compliant (1 point), and Non-Compliant (0 points) [31], differences were discussed with the third investigator (Hao Wu) to reach consistent conclusions [32].

### Data synthesis and analysis

Review Manager 5.4 was used for meta-analyses. Dichotomous data were expressed as risk ratios (RR), risk differences (RD), or odds ratios (OR) and continuous data as mean differences (MD) or standardized mean differences (SMD). We analysed statistical heterogeneity using the chi-square test (P > 0.10 for statistical significance) and quantified using the *I*^2^ statistic. If heterogeneity was not significant (*P* > 0.05, *I*^2^ < 50%), we performed a quantitative data synthesis (meta‐analysis) using a fixed‐effect model. Where heterogeneity was significant (*P* < 0.05), we analysed the cause to determine whether qualitative differences existed. If the clinical heterogeneity was not due to qualitative differences, we applied a random‐effects model [33–35].

### Sensitivity analysis

If necessary, the sensitivity analysis will be used to assess the effect of each study on the random effects model. The sensitivity of the general combined effect of all outcome indicators will be analyzed by the exclusion method. In short, each study will be excluded and the rest will be re-analyzed to determine the stability of the results, which will be considered stable if the combined effects shown have not changed qualitatively.

### Risk of bias across trials

When a meta-analysis contained at least 10 RCTs, the potential risk of bias across trials was examined by the funnel plots and Egger’s test. However, this meta-analysis contained less than 10 RCTs, so the funnel plot will not be considered [36, 37].

### Quality of evidence

Three independent investigators assessed the risk of bias in each included trial using the GRADE (Grading, Evaluation, Development and Evaluation of Recommendations) approach [38–41]. Three levels of bias were used to assess the degree of bias in the trial by two investigators (Gang Luo and Yu Zheng) which included "low risk of bias" (None), "high risk of bias" (Serious), and "unspecified risk of bias" (No). The third investigator (Hao Wu) assessed for downgrade or promotion and divided into four grades: High (⊕⊕⊕⊕/A), Medium (⊕⊕⊕Ο/B), Low (⊕⊕ΟΟ/C) and extremely low (⊕ΟΟΟ/D). The risk of bias was mainly evaluated according to the following eight aspects: (a) Limitations of study design: there is an additional risk of misleading results due to flaws in the design or implementation of randomized controlled trials. (b) Inconsistency: estimates vary widely from study to study, the confidence intervals for each study are very narrow or do not overlap, the p-value for the heterogeneity test is small and the *I*^2^ value is large. (c) Indirect: population differences, intervention differences, outcome measures differences, and indirect evidence comparisons. (d) Imprecision: small sample size means less than 300 events per piece of evidence. (e) Publication bias: Reporting bias and poor robustness of results. (6) Large effect size: The effect size of the study is large and ideal, which can better improve the quality of the study. (f) There is a dose-effect relationship: the dose-effect relationship can describe the relationship between the results more clearly, which is of positive significance for research. (g) Negative bias. Negative bias makes the original bias return to normal, which also has a certain positive significance for research. Evidence will be lowered one level when the first five situations mentioned above occur. If there were bad trials, the evidence would be downgraded by two levels. When the last three situations mentioned above occur, the level of evidence should be appropriately raised and the level of evidence should be comprehensively assessed finally [42].

## Results

### Study identification

**Fig 1:**
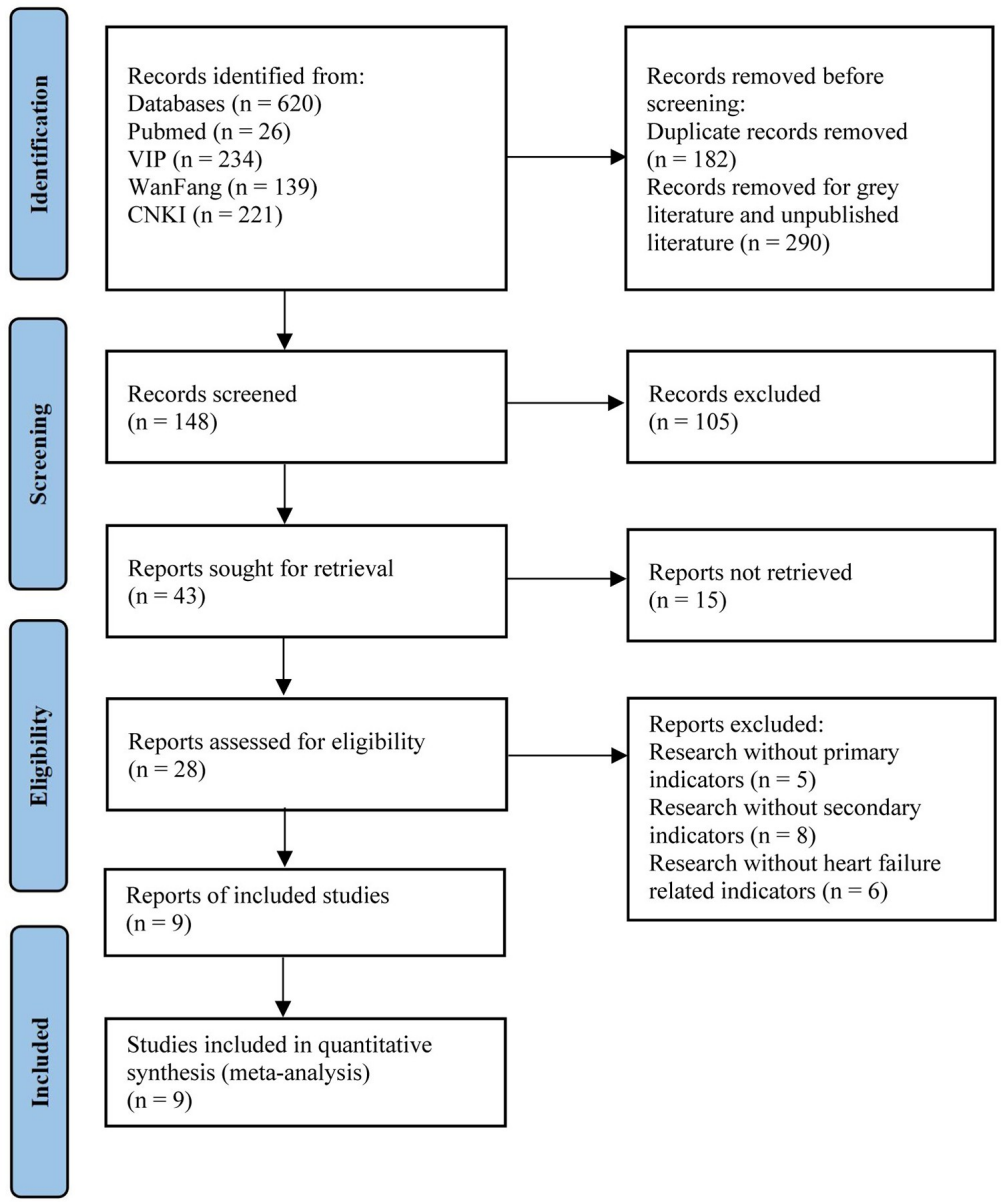
Screen of articles.

The study selection and identification process are shown in Fig 1. Initially, we collected 620 records via databases and the manual literature search, including Pubmed (n = 26), VIP (n = 234), WanFang (n = 139), CNKI (n = 221). Among these, 182 articles for duplication were discarded, and 290 grey literature and unpublished literature were removed. After reviewing the titles and abstracts, 120 articles that were not RCTs were removed. Subsequently, a total of 28 full-text articles were retrieved for further evaluation, of which 19 were excluded for the following reasons: RCTs without outcome indicators (n = 13) and RCTs without HF-related indicators study (n = 6). Finally, 9 RCTs were included in this study. Since this meta-analysis contained fewer than 10 RCTs, funnel diagram were not applicable, and the results of this meta-analysis may be potentially biased.

### Characteristics of the included RCTs

The basic characteristics of the nine included RCTs are shown in Table 2. Overall, all RCTs used a randomized controlled design (one experimental group and one control group), we identified 855 participants from 9 articles with 429 cases in the experimental group and 426 cases in the control group. All the included studies were published in Chinese, and all RCTs had small sample sizes, ranging from 30 to 87 people. The average age of patients participating in the study was 54 to 63 years old. HF was first diagnosed in 6 RCTs [43–48], and coronary heart disease was the first diagnosis in the other 2 RCTs [49, 50], and cardiomyopathy was the first diagnosis in the remaining one, According to the latest definitions and diagnostic criteria for heart failure, combined with the patient’s clinical manifestations and echocardiographic indicators, all patients included in the study met the diagnostic criteria for heart failure [51]. For the intervention, the patients in the experimental group received different doses of Danshen decoction combined with CT (Doses range from 200 mL/bid to 400 mL/bid), and patients in the control group received CT alone. CER was identified as the primary outcome. CF including left ventricular ejection fraction (LVEF) [52], left ventricular end-diastolic dimension (LVEDD), and left ventricular end-systolic diameter (LVESD) [53], and serological indicators including BNP, NT-proBNP, and hs-CRP, were identified as secondary outcomes [54]. The duration of treatment varied from 2 weeks to 2 months.

**Table 2:** The characteristics of the included studies.

Reference	Diagnosis	Gender M/F		Mean age (year)		Sample size (Control/Treatment)	Dose and process of treatment		Clinical outcomes	CER	Course of treatment	Jadad Score
		Control	Treatment	Control	Treatment		Control	Treatment				
(Xu and Kong, 2021)	Heart Failure	18/12	19/11	56.72 ± 2.02	56.81 ± 2.18	30/30	Bisoprolol (10 mg/qd) Benazepril (20 mg/qd) Spironolactone (20 mg/qd)	CT + Danshen decoction (350 mL/bid)	LVEF, LVEDD, LVESD	93.33%	28 day	3
(Wang et al., 2019)	Heart Failure	38/17	36/19	55.6 ± 5.7	56.2 ± 6.2	55/55	CT	CT + Danshen decoction (350 mL/bid)	LVEF, BNP	92.73%	28 day	2
(Wu et al., 2020)	Heart Failure	19/19	20/18	60.14	58.72	38/38	Perindopril (2 mg/qd) Metoprolol (23.75 mg/qd)	CT + Danshen decoction (200 mL/bid)	LVEF, NT-ProBNP	97.37%	28 day	2
(Wei and Yang, 2018)	Heart Failure	19/11	18/12	63–81	62–82	30/30	Furosemide (20 mg/qd) Ramipril (2.5 mg/qd) Betaloc (12.5 mg/bid) Digoxin (0.125 mg/bid) Spironolactone (20 mg/qd)	CT + Danshen decoction (400 mL/qd)	LVEF, BNP	93.33%	14 day	2
(Guo and Zhou, 2021)	Heart Failure	29/27	31/25	62.95 ± 5.97	62.34 ± 5.83	56/56	CT	CT + Danshen decoction (1 dose/qd)	LVEF, LVEDD, NT-proBNP, hs-CRP	89.29%	42 day	3
(Cai et al., 2015)	Heart Failure	38/45	41/46	59.94 ± 12.6	61.32 ± 11.3	83/87	CT	CT + Danshen decoction (200 mL/bid)	LVEF, LVEDD, LVESD	93.10%	15 day	3
(Xue et al., 2019)	Coronary Heart Disease	23/17	22/17	55.83 ± 11.24	54.32 ± 11.39	40/39	CT	CT + Danshen decoction (1 dose/qd)	hs-CRP, NT-proBNP, LEVF, LVESD	92.31%	28 day	2
(Liu, 2018)	Coronary Heart Disease	24/14	23/15	60.34 ± 2.91	59.01 ± 2.13	38/38	Potassium magnesium aspartate (20 mg/bid) Glyceryl trinitrate (10 mg/qd) Indomethacin (10 mg/tid)	CT + Danshen decoction (1 dose/bid)	LVEF	94.74	30 day	2
(Sheng and Shen, 2022)	Cardiomyopathy	27/29	30/26	62.86 ± 6.59	62.59 ± 6.25	56/56	Enalapril (5 mg/bid)	CT + Danshen decoction (400 mL/bid)	LVEF, LVEDD, hs-CRP	87.5%	60 day	3

### Evaluation of methodological quality

**Fig 2:**
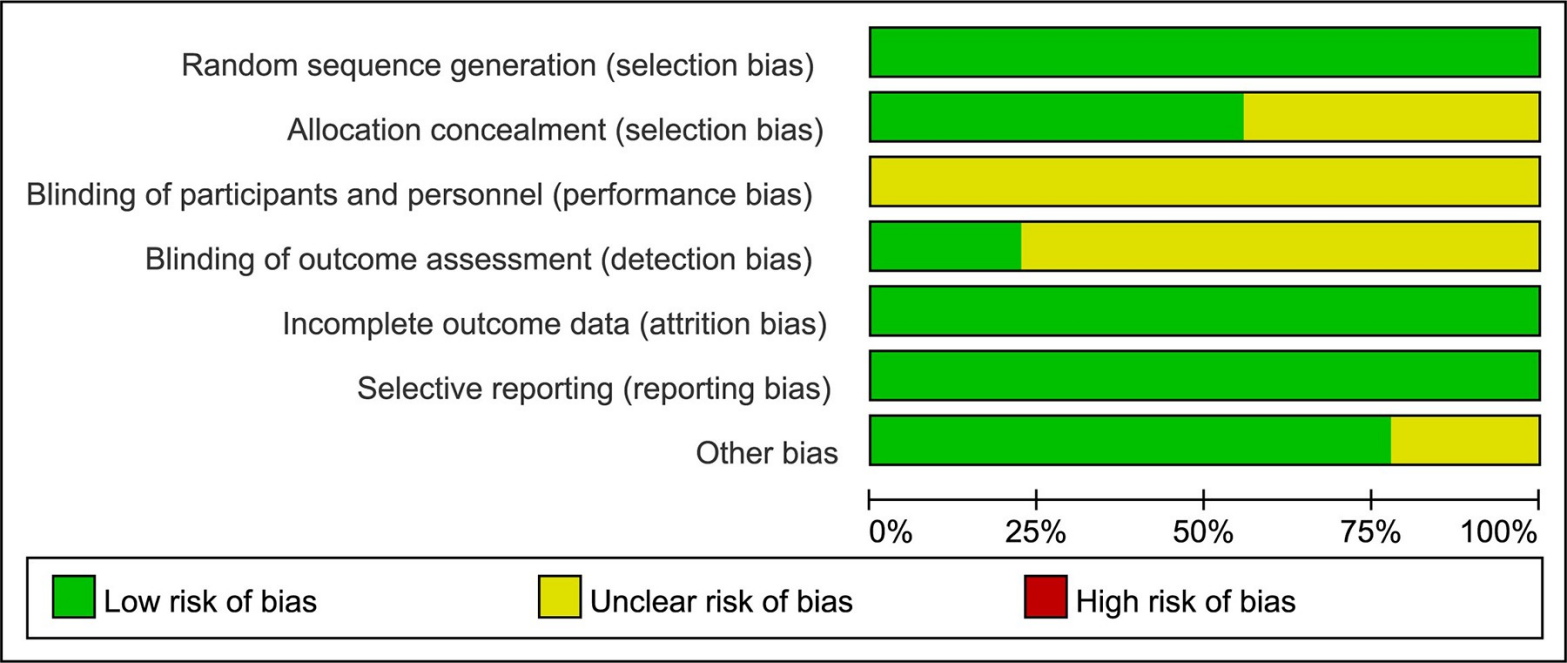
Risk of bias graph.

**Fig 3:**
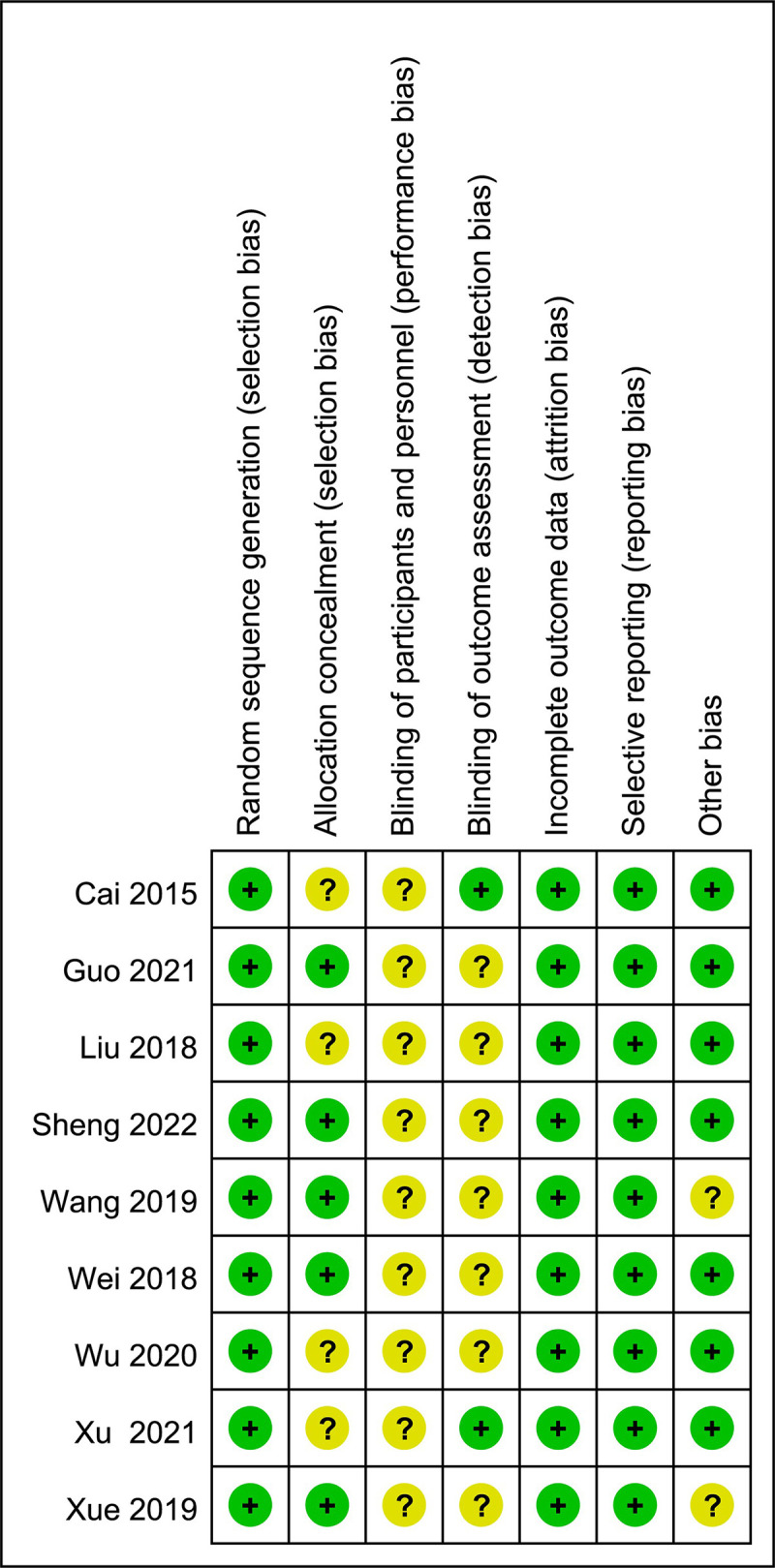
Risk of bias summary.

The methodological quality of the included studies is shown in Figs 2 and 3. Nine RCTs [43–51] mentioned that sequence generation was illustrated by randomization or by random number tables and plots and rated as low risk concerning overall quality. Five RCTs [44–46, 50, 51] mentioned random assignment concealment and analyzed specific implementation measures considered to have low random bias, and the remaining three RCTs [43, 47, 55] did not clearly describe assignment concealment and were defined as unclear. None of the nine RCTs described the blinding of participants and personnel, the performance bias was considered unclear. The blinding of outcome assessment in two RCTs [43, 48] was well described and considered to have low random sequence bias, while the remaining RCTs were poorly defined as unclear. All nine RCTs completely reported the CER and LVEF as outcomes, considered to have low attrition bias and reporting bias. Other biases (including but not limited to selection bias, information bias, confounding bias, etc.) are considered to exist in two RCTs [45, 50] due to unclear descriptions. Taken together, all RCTs were considered not to be at high risk of bias. Table 2 and S3 Table were presented for the Jadad scores of all RCTs.

### Primary outcome

#### CER

**Fig 4:**
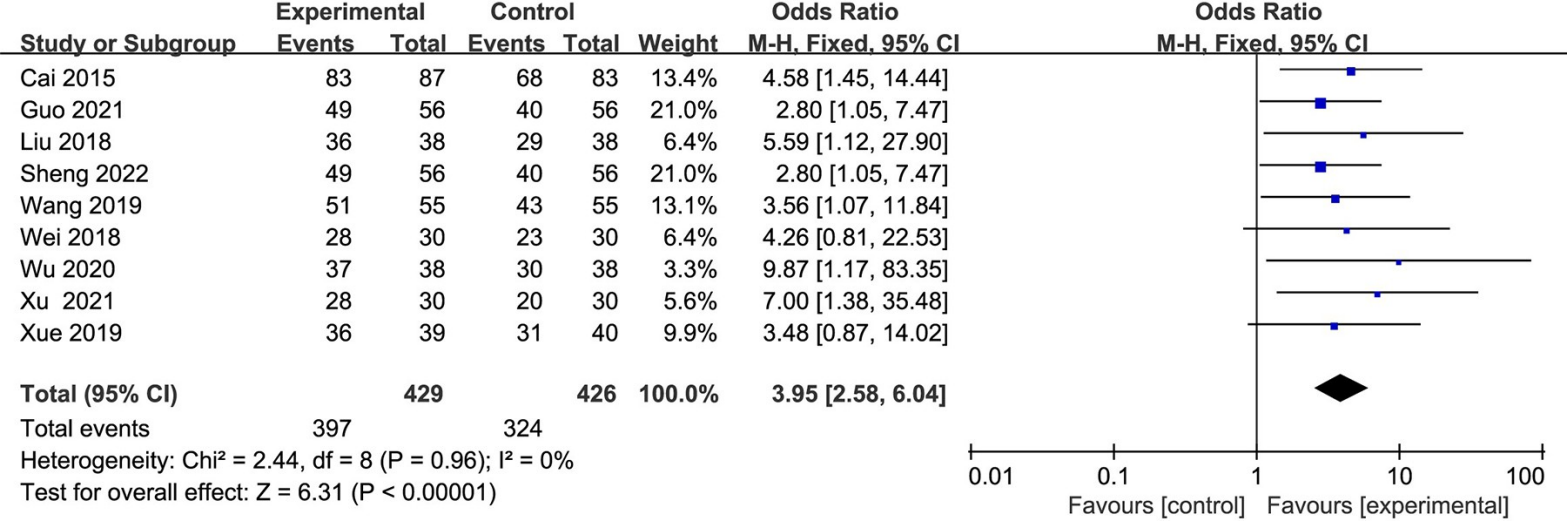
Forest plots showing a significant improvement in the CER in the experiment group compared with the control group.

A total of nine studies [43–51] with 855 patients, including 426 cases in the control group and 429 cases in the experimental group. Compared with the CT alone, the CER (%) has been significantly improved due to the combination of Danshen decoction and CT. There was statistical homogeneity for this outcome (*I*^2^ = 0%), and the fixed-effects model was applied (MD = 3.95, 95% CI [2.58, 6.04], *P* < 0.00001) (Fig 4).

### Second outcomes

#### LVEF

**Fig 5:**
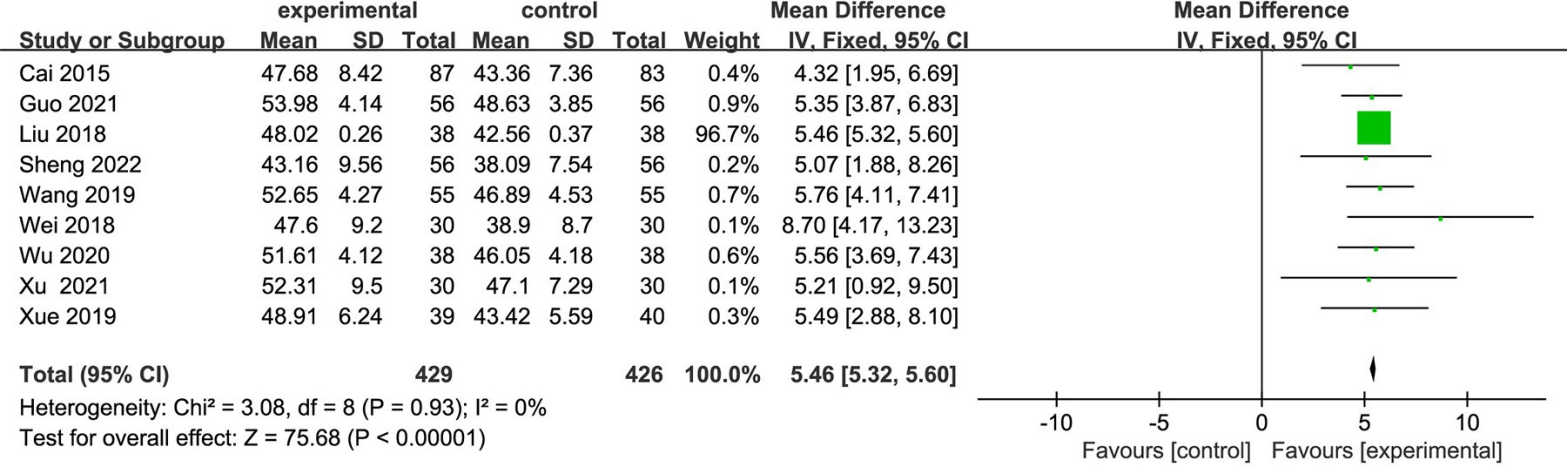
Forest plots showing a significant improvement in the LVEF in the experiment group compared with the control group.

A total of nine studies [43–51] with 855 patients, including 426 cases in the control group and 429 cases in the experimental group. Compared with CT alone, the LVEF (%) was significantly improved due to the combination of Danshen decoction and CT. There was statistical homogeneity for this outcome (*I*^2^ = 0% < 50%), and the fixed-effects model was applied (MD = 5.46, 95% CI [5.32, 5.60], *P* < 0.00001) (Fig 5).

#### LVEDD

**Fig 6:**
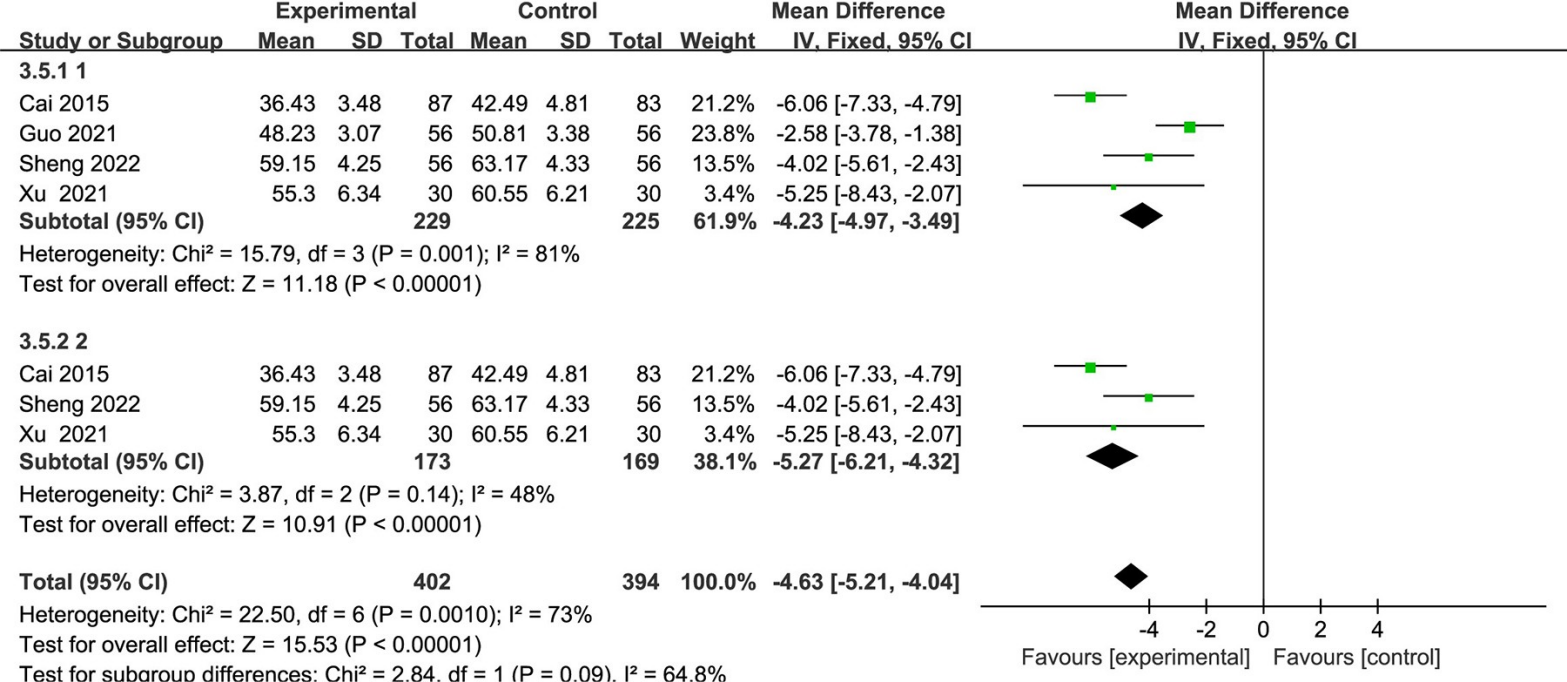
Forest plots showing a significant improvement in the LVEDD in the experiment group compared with the control group.

A total of four studies [43, 44, 48, 51] with 172 patients, including 86 in the control group and 86 in the experimental group. Compared with CT alone, the LVEDD (mm) was significantly decreased due to the combination of Danshen decoction and CT, heterogeneity existed across the studies (*I*^2^ = 81%, *P* = 0.001), a random-effects model was applied (MD = -4.39, 95% CI [-6.22, -2.55], *P* < 0 .00001). In order to analyze the reasons for the high heterogeneity, a subgroup analysis was performed after checking the specific information of the RCTs. After rechecking the specific information of the included research subjects, it was found that the HF grades of the subjects in three RCTs [43, 48, 51] were grades II to III, while the HF grades of the subjects in the one RCT [44] were grades III to IV, the cause of high clinical heterogeneity is considered to be related to the severity of the disease, after excluding this study, there was statistical homogeneity for this outcome (*I*^2^ = 48%), and the fixed-effects model was applied (MD = -5.27, 95% CI [-6.21, -4.32], *P* < 0.00001) (Fig 6).

#### LVESD

**Fig 7:**
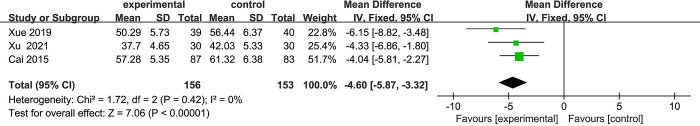
Forest plots showing a significant improvement in the LVESD in the experiment group compared with the control group.

A total of three studies [43, 48, 50] with 309 patients, including 153 in the control group and 156 in the experimental group. Compared with CT alone, the LVESD (mm) was significantly decreased due to the combination of Danshen decoction and CT. There was statistical homogeneity for this outcome (*I*^2^ = 0%), and the fixed-effects model was applied (MD = -4.60, 95% CI [-5.87, -3.32] *P* < 0.00001) (Fig 7).

#### BNP

**Fig 8:**
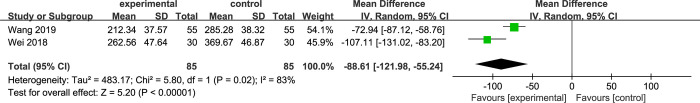
Forest plots showing a significant improvement in the BNP in the experiment group compared with the control group.

A total of two studies [45, 46] with 170 patients, including 85 in the control group and 85 in the experimental group. Compared with CT alone, BNP (pg/mL) was significantly decreased due to the combination of Danshen decoction and CT, and heterogeneity existed across the studies (*I*^2^ = 83%, *P* < 0.00001), the random-effect model was applied (MD = -88.61, 95% CI [-121.98, -55.24], *P* < 0.00001) (Fig 8). There were significant differences in the age and course of treatment of the subjects of this clinical study, which were initially considered to be clinical heterogeneity.

#### NT-proBNP

**Fig 9:**
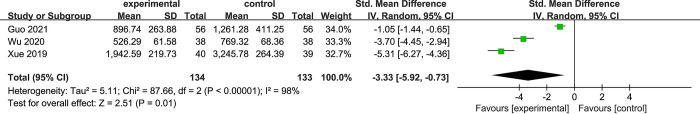
Forest plots showing a significant improvement in the NT-proBNP in the experiment group compared with the control group.

A total of 3 studies [44, 47, 50] involving 267 patients, including 133 cases in the control group and 134 cases in the experimental group. Compared with CT alone, the NT-proBNP (pg/mL) was significantly decreased due to the combination of Danshen decoction and CT, and heterogeneity existed across the studies (*I*^2^ = 98% < 50%, *P* < 0.00001), the random-effect model was applied (SMD = -3.33, 95% CI [-5.92, -0.73], *P* = 0.01) (Fig 9). Sensitivity analysis showed that no obvious heterogeneity was found after removing any study. There were differences in the course of disease, medication time and dose of the subjects in the RCTs included in this group, which were initially considered to be clinical heterogeneity.

#### hs-CRP

**Fig 10:**
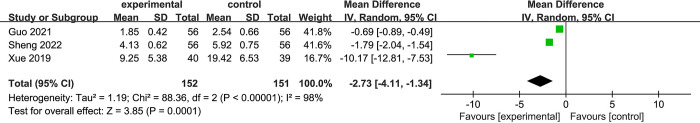
Forest plots showing a significant improvement in the hs-CRP in the experiment group compared with the control group.

A total of three studies [44, 50, 51] involving 303 patients, including 151 cases in the control group and 152 cases in the experimental group. Compared with CT alone, the hs-CRP (mg/L) was significantly decreased due to the combination of Danshen decoction and CT, and heterogeneity existed across the studies (*I*^2^ = 98% > 50%, *P* < 0.00001), the random-effect model was applied (MD = -2.73, 95% CI [-4.11, -1.34], Z = 3.85, *P* = 0.0001) (Fig 10). There were significant differences in the first diagnosis and disease course of the three RCTs in this group, and they could not be improved according to the acute and non-acute subgroups of the disease, which was initially considered to be clinical heterogeneity.

### Quality of evidence

In this study, the quality of evidence for all included RCTs was downgraded by one grade because the risk of methodological bias was unclear. With the exception of CER, LVEF, LVESD and hs-CRP, the number of patients included in LVEDD, BNP and NT-probNP was no more than 300, so the quality of evidence for these outcomes was downgraded by one grade. The quality of the evidence suggests that the escalation did not apply to any outcome. Overall, the quality of evidence was moderate for CER, LVEF, LVESD and hs-CRP, and low for LVEDD, BNP and NT-proBNP (Table 3).

**Table 3:** GRADE evidence profile of clinical efficacy and safety.

Outcomes (Trials)	Quality assessment								Interventions		Clinical efficacy		Quality
	Risk of bias	Inconsistency	Indirectness	Imprecision	Publication bias	The effect value is very large	Dose effect relationship	Negative bias	Danshen decoction + CT	CT	95% CI	Adverse reaction report	
CER (9)	Serious ^a^	No	No	No	None ^c^	Serious^d^	No	None	429/855 (50.2%)	426/855 (49.8%)	2.58 to 6.04	None	⊕⊕⊕O / B
LVEF (9)	Serious ^a^	No	No	No	None ^c^	Serious^d^	No	None	429/855 (50.2%)	426/855 (49.8%)	5.32 to 5.60	None	⊕⊕⊕O / B
LVEDD (4)	Serious ^a^	No	No	Serious^b^	None ^c^	No	No	None	173/342 (50.6%)	169/342 (49.4%)	- 6.60 to—3.70	None	⊕⊕OO / C
LVESD (3)	Serious ^a^	No	No	Serious^b^	None ^c^	Serious^d^	No	None	156/309 (50.5%)	153/309 (49.5%)	- 5.87 to—3.32	None	⊕⊕⊕O / B
BNP (2)	Serious ^a^	No	No	Serious^b^	None^c^	No	No	None	85/170 (50.0%)	85/170 (50.0%)	- 121.98 to—55.24	None	⊕⊕OO / C
NT-proBNP (3)	Serious ^a^	No	No	Serious^b^	None^c^	No	No	None	134/267 (50.2%)	133/267 (49.8%)	- 5.92 to—0.73	None	⊕⊕OO / C
hs-CRP (3)	Serious ^a^	No	No	Serious^b^	None^c^	Serious^d^	No	None	152/303 (50.2%)	151/303 (49.8%)	- 4.11 to—1.34	None	⊕⊕⊕O / B

Notice:

^a^ Most domain had unclear methodological bias risk. Evidence was rated down by only one level.

^b^ The sample size for each indicator was less than 100 cases, and the evidence was rated down by one level.

^c^ The publication bias was presented. The results had good robustness. Not rating down.

^d^ Large sample size and significant results. Rating increase.

## Discussion

Overall, this study has the following characteristics: (a) This is a PROSPERO-registered meta-analysis, and the analysis method will be published and updated in time for further research. (b) Electronic and manual retrieval methods were used to ensure that the included RCTs are relevant for meta-analysis. (c) Our confidence in the findings was further increased by performing robust sensitivity analyses and reliable subgroup analyses.

As revealed in this meta-analysis, CER is a comprehensive evaluation index derived from multiple clinical indicators and patient subjective rating scales, and was defined as the primary outcome in this study [56]. All the RCTs included in the meta-analysis obtained satisfactory CERs from the data of their respective studies, and achieved statistically significant heterogeneity, which means that the patient’s various heart failure indicators have been greatly improved and affirmed the effect of Danshen decoction combined with CT in the treatment of HF [57]. In terms of objective indicators, cardiac color doppler ultrasound is mainly used to evaluate the changes of CF in patients. Among the data measured by multiple cardiac color doppler ultrasound, LVEF is considered to be the most valuable indicator. In this study, LVEF was included in all RCTs, and the first diagnosis of these patients included HF, myocardial infarction and coronary heart disease. The results showed that Danshen decoction combined with CT could significantly improve the LVEF of patients to treat HF. In addition, LVEDD and LVESD in each experimental group as cardiac color doppler ultrasound observation data were significantly reduced under the treatment of Danshen decoction combined with CT, and showed statistically significant heterogeneity, which further proved the clinical effect of Danshen decoction. BNP and NT-proBNP reflect the severity of HF, while hs-CRP reflects the possible inflammatory response in the patient. The results of the meta-analysis showed that all serological indexes could be significantly reduced under the treatment of Danshen decoction combined with CT, but the heterogeneity was obvious, and no statistically significant heterogeneity could be obtained through sensitivity analysis and subgroup analysis. The severity of the disease in RCTs was considered to be the cause of the high heterogeneity, and more randomized controlled trials facilitated more satisfactory heterogeneity results.

TCM is characterized by multiple pharmacodynamic material basis, multiple therapeutic targets and low side effects [55, 58]. A large number of pharmacological studies have verified that Danshen decoction has the functions of anti-myocardial fibrosis, protecting myocardial cells, maintaining the ultrastructure of the heart, and improving coronary blood flow [59, 60], however, there are few reports in the clinic studies that significant adverse reactions of Danshen decoction occurred during the use of patients. This meta-analysis verifies that Danshen decoction as a complementary therapy has a definite effect on the improvement of HF symptoms, as well as a significant effect on patients with different cardiovascular disease-induced symptoms of HF, and none of the included RCTs reported adverse effects. Therefore, Danshen decoction is an effective and safe complementary therapy for HF with great clinical application prospects.

### Limitations and suggestions

#### Limitations

First of all, among the RCTs included in this study, only 1 RCT reported no adverse reactions during the use of Danshen decoction, and the remaining 8 RCTs reported no adverse reactions. As far as we know, the safety evaluation of drugs includes toxicological studies, contraindications, precautions, drugs in special groups, drug interactions and adverse reactions. At present, the toxicology studies of Danshen decoction show that no obvious side effects are found in acute and chronic toxicological experiments, and Danshen decoction is a low-toxic prescription [61]. By reviewing the clinical studies of Danshen decoction, there were no obvious adverse effects of Danshen decoction as adjuvant therapy or combination therapy, and any contraindications and special population were found. Therefore, We have a positive assessment of the security of Danshen decoction. However, more rigorous evidence-based medical evidence and more extensive clinical studies are still needed to demonstrate the safety of Danshen decoction [62]. However, systematic reports of adverse reactions and other safety assessments are still lacking in clinical studies. Secondly, in terms of RCT quality evaluation, only 2 of the 9 RCTs had higher Jadad scores and GRADE grades, and the remaining RCTs had more or less research bias, for example, the logic of experimental design, the randomness of experimental object selection, comprehensiveness of experimental result selection and avoidance of other bias cannot be fully reflected in RCTs [63]. All the participants in the included studies were Chinese, and the uncertainty of racial and regional differences were high [64]. Finally, as a classic TCM formulation, Danshen decoction currently does not have the production process and quality control standards formulated by the International Organization for Standardization, therefore, during the clinical research, there may be differences in the Danshen decoction prepared by different hospitals or individuals, and there is no RCT study using Danshen decoction alone as the treatment of the experimental group, which further increases the limitations of this study [65, 66].

#### Suggestions

In order to improve the strength of evidence for the treatment of cardiovascular diseases with Danshen decoction, future clinical research should focus on the following aspects [67]: (a) With the transformation of the human disease spectrum and modern medical model, Danshen decoction has been more widely used in the clinical treatment of HF in the form of complementary therapy in the future, the influence of Danshen decoction on the long-term prognosis of the disease and adverse reactions should be clarified in order to further verify its effectiveness and safety. (b) More multi-center, larger randomized controlled trials are required to improve the quality of clinical research and the strength of evidence, rigorous random allocation methods and blinding operations are needed to be adopted, wider research participants and more comprehensive outcome measures should be selected [68]. (c) The quality standard of Danshen decoction should be determined to reduce the clinical bias in drug intervention, the analysis of active components of Danshen decoction needs to be deeply studied, and the research on the mechanism of action of Danshen decoction on HF and the research on pharmacodynamic substances should be widely carried out [69].

## Conclusions

Danshen decoction combined with conventional treatment significantly improved the clinical effective rate, cardiac function, and serological indicators in heart failure patients, and there were no adverse events reported due to this combination therapy, indicating that Danshen decoction as an adjuvant therapy is a promising treatment option for heart failure. However, given the limitations of RCTs in this study, more rigorous, larger and multicenter randomized clinical trials are guaranteed to further evaluate the efficacy and safety of Danshen decoction for patients with heart failure.

## Supporting information

S1 Table PRISMA 2020 checklist. (DOCX)

S2 Table Search strategy in PubMed. (DOCX)

S3 Table Improved Jadad score. (DOCX)

## Abbreviations

BNP: Brain natriuretic peptide
CER: Clinical effective rate
CF: Cardiac function
CI: Confidence intervals
CRP: C-reactive protein
CT: Conventional Treatment
CVD: Cardiovascular diseases
HF: Heart failure
hs-CRP: Hypersensitive C-reactive protein
MD: mean difference
NT-proBNP: N-terminal pro-B type natriuretic peptide
OR: Odds ratios
PRISMA: The Preferred Reporting Items for Systematic Reviews and Meta-Analyses
RCT: Randomized controlled trial
RD: Risk differences
RR: Risk ratios
SMD: Standardized mean difference
TCM: Traditional Chinese medicine
WHO: World Health Organization
