# Supplementary material for: Clinical effect of Danshen decoction in patients with heart failure: A systematic review and meta-analysis of randomized controlled trials
Source: PLoS One. 2023 May 5;18(5):e0284877. doi: 10.1371/journal.pone.0284877 (PMC10162557; doi:10.1371/journal.pone.0284877)
Supplement: S2 Table — (DOCX) [file pone.0284877.s002.docx]

**Table S2. Search Strategy in PubMed.**

|  | **Search Strategy in PubMed** |
| --- | --- |
| **Search** | **Query** |
| #1 | Heart Failure[MeSH Terms] |
| #2 | Heart failure[Title/Abstract] |
| #3 | Cardiac failure[Title/Abstract] |
| #4 | Heart decompensation[Title/Abstract] |
| #5 | Heart dysfunction[Title/Abstract] |
| #6 | Cardiac dysfunction[Title/Abstract] |
| #7 | Ventricular dysfunction[Title/Abstract] |
| #8 | Heart dificiency[Title/Abstract] |
| #9 | Cardiac dificiency[Title/Abstract] |
| #10 | Heart insufficiency[Title/Abstract] |
| #11 | Cardiac insufficiency[Title/Abstract] |
| #12 | #1 OR #2 OR #3 OR #4 OR #5 OR #6 OR #7 OR #8 OR #9 OR #10 OR #11 |
| #13 | Danshen Decoction[MeSH] |
| #14 | Danshen Decoction[Title/Abstract] |
| #15 | Danshen Yin[Title/Abstract] |
| #16 | #13 OR #14 OR #15 |
| #17 | Randomized Controlled Trials as Topic[Mesh] |
| #18 | Randomized Controlled Trial[Publication Type] |
| #19 | Clinical Controlled experiment[Publication Type] |
| #20 | Clinical Observation[Publication Type] |
| #21 | Danshen decoction[Title/Abstract] |
| #22 | Cardiovascular System[Mesh] |
| #23 | Coronary heart disease[Mesh] |
| #24 | heart failure[Mesh] |
| #25 | ischemia-reperfusion injury[Publication Type] |
| #26 | angina pectoris[Mesh] |
| #27 | heart valve disease[Mesh] |
| #28 | hypertension[Title/Abstract] |
| #29 | pericardial disease[Title/Abstract] |
| #30 | endocarditis[Title] |
| #31 | cardiac arrest and sudden cardiac death[Title/Abstract] |
| #32 | #17 OR #18 OR #19 OR #20 OR #21 OR #22 OR #23 OR#24 OR #25 OR #26 OR #27 OR #28 OR #29 OR #30 OR #31 |
| #33 | #12 AND #16 AND #32 |
