# Supplementary material for: Clinical effect of Danshen decoction in patients with heart failure: A systematic review and meta-analysis of randomized controlled trials
Source: PLoS One. 2023 May 5;18(5):e0284877. doi: 10.1371/journal.pone.0284877 (PMC10162557; doi:10.1371/journal.pone.0284877)
Supplement: S3 Table — (DOCX) [file pone.0284877.s003.docx]

**Table S3. Improved Jadad score.**

| Reference | Random sequence generation | Randomize hide | Blind method | Retreat and withdrawal | Total score |
| --- | --- | --- | --- | --- | --- |
| (Xu and Kong, 2021) | 2 | 1 | 0 | 0 | 3 |
| (Wang et al., 2019) | 1 | 1 | 0 | 0 | 2 |
| (Wu et al., 2020) | 1 | 1 | 0 | 0 | 2 |
| (Wei and Yang, 2018) | 1 | 1 | 0 | 0 | 2 |
| (Guo and Zhou, 2021) | 2 | 1 | 0 | 0 | 3 |
| (Cai et al., 2015) | 2 | 1 | 0 | 0 | 3 |
| (Xue et al., 2019) | 1 | 1 | 0 | 0 | 2 |
| (Liu, 2018) | 1 | 1 | 0 | 0 | 2 |
| (Sheng and Shen, 2022) | 2 | 1 | 0 | 0 | 3 |
